# The effects of using a lateral wedge insole on knee loading during ascending and descending stairs

**DOI:** 10.1186/1757-1146-5-S1-P11

**Published:** 2012-04-10

**Authors:** Amneh Alshawabka, Richard Jones, Sarah Tyson

**Affiliations:** 1 Centre for Health Sciences Research, University of Salford, Salford, Greater Manchester, M6 6PU, UK

## Background

Stair climbing demands, as compared to walking level, a greater range of motion in the lower extremity accompanied by about six times more load on knee joint [1]. Consequently, pain while climbing stairs is the first complaint in patients with knee osteoarthritis (OA) [2]. The use of lateral wedge insoles aims to decrease medial knee compartment loading by reducing the peak external knee adduction moment (EKAM) during walking [4]. The purpose of this study is to assess the biomechanical effects of wearing lateral wedge insoles on EKAM during stair climbing in elders with and without knee OA.

## Methods

Thirty healthy subjects (21 females, 9 males; age (45.7±5.6 years)) and eight patients with mild knee OA (5 females, 3 males; age (47.3±3)) participated in the study. Subjects performed five trials of step-over-step stairs ascent and descent. Two conditions were investigated: (a) control (Standard shoe) (b) 5 degrees Salford Insole lateral wedge (LW) insoles. Kinematic and kinetic data were collected for the lower extremity using a motion capture system (QTM^Tm^) and two force plates (AMTI force platform stairway). Repeated measures ANOVA and Friedman’s ANOVA were used for statistical analysis.

## Results

During ascending stairs, LW significantly reduced the EKAM in early stance (p<.05) and the knee adduction angular impulse (KAAI) (p<.05). Similarly, the EKAM and KAAI had been significantly reduced (p<.05) while wearing LW during descending stairs. Both groups had significantly greater degree of subtalar eversion with LW than in the control condition (Table 1).

**Table 1:** 1st EKAM peak, KAAI and Subtalar eversion angle results for healthy and OA Subjects during ascending (AS) and descending (DS) stairs.

Parameters		Mean ± (SD)		Mean ± (SD)	
		Control (Healthy)	LW (Healthy)	Control (OA)	LW (OA)
1^st^ peak EKAM (Nm/Kg)	AS	.385 (.15 )	.357 (.14)	.394 (.13)	.366 (.12)
	
	DS	.408 (.11)	.388 (.10)	.364 (.06)	.334 (.05)

KAAI (Nm/Kg/s)	AS	.228 (.15)	.207 (.14)	.189 (.06)	.174 (.06)
	
	DS	.228 (.08)	.212 (.08)	.204 (.04)	.186 (.04)

Subtalar peak eversion (degrees)	AS	-5.71 (2.4)	-6.1 (2.9)	-4.41 (.62)	-4.82 (.70)
	
	DS	-6.36 (2.3)	-7.06 (2.5)	-4.42 (1.2)	-5.11 (1.5)

## Conclusions

Lateral wedge insoles consistently reduced the overall magnitude of EKAM during ascending and descending stairs which has been strongly correlated to decreasing medial compartment loading at the knee joint. Thus, these results give the first indication that that lateral wedge insoles may be useful in decreasing pain levels for patients with knee OA during stair climbing. Further long-term studies are warranted.
